# 以血小板减少为首发临床表现的成人植物固醇血症8例临床分析

**DOI:** 10.3760/cma.j.cn121090-20240710-00257

**Published:** 2025-03

**Authors:** 艳洁 胡, 文兰 陈, 梅 薛, 雅洁 丁, 恒 梅, 雅丹 王

**Affiliations:** 华中科技大学同济医学院附属协和医院血液科，武汉 430022 Department of Hematology, Tongji Medical College Affiliated Union Hospital, Huazhong University of Science and technology, Wuhan 430022, China

**Keywords:** 植物固醇血症, ABCG5和ABCG8基因突变, 平均血小板体积, 依折麦布, Phytosterolemia, ABCG5 and ABCG8 gene mutations, Mean platelet volume, Ezetimibe

## Abstract

**目的:**

分析以血小板减少为首发临床表现的成人植物固醇血症患者的临床特征。

**方法:**

对2020年12月至2023年12月在华中科技大学同济医学院附属协和医院就诊的8例成人植物固醇血症患者进行回顾性分析。

**结果:**

①8例植物固醇血症患者中男2例（25％）、女6例（75％），中位诊断年龄55（29～66）岁。发现血小板减少到确诊的中位病程为10（0.2～50）年。②8例患者的平均血小板体积（MPV）和大血小板比例（LPR）明显高于健康对照组（30例成人志愿者）和原发免疫性血小板减少症（ITP）患者（20例），外周血涂片平均血小板直径（MPD）和大血小板（直径>4 µm）比例明显高于健康对照和ITP对照组（*P*<0.01）。③5例患者接受低植物固醇饮食和依折麦布治疗，血中谷固醇和菜油固醇含量下降，血红蛋白和血小板计数上升，血小板体积下降。

**结论:**

以血小板减少为首发临床表现的成人植物固醇血症易被误诊；伴溶血性贫血、脾脏肿大，外周血涂片见巨大血小板和口形红细胞增多以及脂黄瘤是诊断该病的重要依据。限制饮食中植物固醇摄入和接受依折麦布治疗可有效降低血中植物固醇含量，改善血液系统异常。

## Abstract (en)

**Objective:**

To analyze the clinical characteristics of adult patients with phytosterolemia presenting with thrombocytopenia as the initial manifestation.

**Methods:**

A retrospective analysis was conducted on eight adult patients with phytosterolemia who visited Union Hospital, Tongji Medical College, Huazhong University of Science and Technology, from December 2020 to December 2023.

**Results:**

① The participants consisted of 2（25％）male and 6（75％）female patients, with a median age at diagnosis of 55 years（range: 29–66 years）. The median duration from the discovery of thrombocytopenia to diagnosis was 10 years（range: 0.2–50 years）. ② Compared with the normal control group（30 healthy adult volunteers）and the immune thrombocytopenia（ITP）control group（20 patients with ITP）, patients with phytosterolemia exhibited significantly higher mean platelet volume and large platelet ratio. Peripheral blood smears revealed that the mean platelet diameter and the proportion of large platelets（diameter> 4 µm）were significantly higher in patients with phytosterolemia than those in the normal and ITP control groups（*P*<0.01）. ③After a low-plant-sterol diet and ezetimibe treatment, five patients demonstrated decreased serum sitosterol and campesterol levels, increased hemoglobin concentration and platelet counts, and reduced platelet volume.

**Conclusion:**

Adult-onset phytosterolemia presenting with thrombocytopenia as the initial manifestation is prone to misdiagnosis. The presence of hemolytic anemia, splenomegaly, increased large platelets and schistocytes on peripheral blood smears, and xanthomas are crucial diagnostic indicators. Restricting dietary plant sterol intake and using ezetimibe to inhibit sterol absorption effectively lowers serum plant sterol levels and improves hematological abnormalities.

植物固醇血症（Phytosterolemia）是一种罕见的常染色体隐性遗传代谢性疾病，由ATP结合盒亚家族G成员5或8（ABCG5或ABCG8）基因发生纯合突变或复合杂合突变引起编码的固醇转运蛋白功能丧失，植物固醇和胆固醇吸收增加、排泄减少，导致植物固醇（包括谷固醇、豆固醇和菜油固醇等）在血液中大量蓄积。其临床表现为动脉粥样硬化、早发型冠心病、皮肤和肌腱的脂黄瘤、关节炎等；此外，25％～35％的患者可出现血液学异常（溶血性贫血、口形红细胞或靶形红细胞、大血小板减少症）和脾肿大[1]–[3]。据估计，该病的患病率约为20万分之一，但到目前为止，全球仅报道200多例患者（中国100余例），这可能由于该病临床症状多样，导致漏诊误诊严重[4]–[5]。本研究对我院收治的以血小板减少为首发临床表现的8例成人植物固醇血症患者的诊疗情况进行回顾性分析。

## 病例与方法

一、病例及健康对照组

本项回顾性研究纳入2020年12月至2023年12月就诊于华中科技大学同济医学院附属协和医院并诊断为植物固醇血症的8例成人患者。本研究获得华中科技大学同济医学院附属协和医院伦理委员会批准（2024伦审字0369号）。植物固醇血症诊断标准：①血浆谷固醇浓度>10 mg/L；②NGS和一代测序验证具有ABCG5和（或）ABCG8基因突变（包括纯合突变/复合杂合突变/双重杂合突变）；③排除遗传性家族高胆固醇血症、原发免疫性血小板减少症（ITP）、Evans综合征等疾病。

ITP组（20例）和健康对照组（30例）均为本院同期就诊人群及健康成人志愿者，其性别和年龄构成比与植物固醇组相匹配。

二、临床资料

通过查阅病历资料和电话随访收集患者性别、年龄、病史、家族史，血常规、血脂、血浆血小板生成素（TPO）浓度、双侧颈动脉、椎动脉、腹主动脉、双肾动脉、双下肢动脉和肝脾B超等检查结果，治疗经过和转归。

三、NGS检查方法

采用探针捕获技术，首先以外周血样本提取的DNA为材料，进行片段化和末端修复、加接头、PCR扩增等步骤制备预文库；其后采用带有生物素标记的寡核苷酸探针与预文库进行杂交；再使用链霉亲和素磁珠与探针结合，从而捕获目标区域；最后经过PCR富集得到最终捕获文库。捕获文库采用基因测序仪Illumina Nextseq AR550进行高通量测序。对于测序数据，采用生物信息学软件判读相关基因中是否存在遗传性的致病基因突变。若有导致血小板减少的阳性突变，进行一代验证和家系验证。

四、植物固醇含量检测

分离患者外周血EDTA抗凝血浆，采用气相色谱-质谱分析仪（GCMS-7890/5975，美国Agilent公司产品）检测血浆固醇水平。以5α-胆甾烷为内参照，通过数据分析仪设备进行图像采集。谷固醇的定量下限为0.3 mg/L，批内、批间变异系数分别为3.94％、15.8％。菜油固醇定量下限为0.25 mg/L，批内、批间变异系数分别为4.37％、12.47％。

五、外周血血小板直径测量

选取植物固醇血症、ITP患者和健康志愿者的外周血涂片进行瑞士吉姆萨染色，在光学显微镜下利用DI-60图像分析系统（日本SYSMEX公司）对100个血小板进行直径测量，分析计算平均血小板直径（MPD）以及大血小板（直径4～8 µm）、巨大血小板（直径>8 µm）的比例。

六、统计学处理

采用SPSS 25.0进行数据分析。计量资料以“均数±标准差”表示，两组间比较采用独立样本*t*′检验。计数资料用构成比或率表示。以*P*<0.05为差异有统计学意义。

## 结果

一、患者及对照组基本资料

8例植物固醇血症患者中位年龄55（29～66）岁，男2例（25％），女6例（75％）。从发现血小板减少到确诊的中位病程为10（0.2～50）年。中位HGB 110（99～156）g/L，其中6例轻度贫血，机测中位血小板计数76（23～122）×10^9^/L。低密度脂蛋白胆固醇（LDL-C）升高4例，胆固醇升高4例，甘油三酯升高3例，1例高密度脂蛋白胆固醇（HDL-C）下降，所有患者谷固醇和菜油固醇含量均升高。5例患者进行了血浆TPO检测，结果均在正常范围内。脂黄瘤3例，均位于面部（睑黄瘤、鼻根部结节黄瘤、面部黄色肉芽肿各1例），动脉粥样硬化3例，关节疼痛2例，脾大7例（其中4例已行脾切除术），有轻微出血表现2例。患者资料详见表1。

**表1:** 8例植物固醇血症患者的临床特征

例号	性别	年龄（岁）	病程（月）	曾使用药物	HGB（g/L）	PLT（×10^9^/L）	LDL-C（mmol/L）	HDL-C（mmol/L）	总胆固醇（mmol/L）	甘油三酯（mmol/L）	谷固醇（mg/L）	菜油固醇（mg/L）	TPO浓度（µg/L）	脂黄瘤	动脉粥样硬化	关节疼痛	脾大	出血
1	女	29	17	糖皮质激素，中药	118	79	3.07	1.62	5.33	0.89	13.46	13.62	19	无	无	无	有	无
2	女	50	23	无	104	61	2.59	1.73	4.85	1.16	45.1	29.83	19	无	有	有	有	无
3	女	56	10	糖皮质激素，TPO，中药	99	23	2.75	1.35	4.08	0.85	50.04	21.41	26	睑黄瘤	有	有	有	牙龈
4	女	66	50	糖皮质激素	104	66	3.11	1.35	5.19	1.35	31.9	12.23	48	面部黄色肉芽肿	有	无	有^a^	无
5	女	48	10	糖皮质激素，达那唑	116	97	3.67	1.2	5.66	1.35	71.9	29.38	40	无	/	无	有^a^	无
6	男	59	2	无	156	77	2.48	1.27	4.51	2.14	49.52	31.24	/	无	/	–	无	无
7	男	57	2	糖皮质激素	152	122	4.41	0.85	5.7	2.31	56.32	20.74	/	无	/	–	有^a^	无
8	女	53	10	糖皮质激素	103	74	5.36	1.37	7.63	2.29	69.25	24.23	/	鼻根部脂黄瘤	/	–	有^a^	鼻出血

**注** LDL-C：低密度脂蛋白胆固醇；HDL-C：高密度脂蛋白胆固醇；TPO：血小板生成素；生物参考值：LDL-C 2.7～3.1 mmol/L，HDL-C 1.16～1.42 mmol/L，总胆固醇<5.2 mmol/L，甘油三酯<1.7 mmol/L，谷固醇0～5 mg/L，菜油固醇0～7 mg/L，TPO 0～196 µg/L；^a^脾已切除；/：不详

健康对照组中位年龄49（14～79）岁，男8例（27％），女22例（73％）。ITP组中位年龄57（15～87）岁，男6例（30％），女14例（70％）。

二、基因突变特点和家系特点

8例植物固醇血症患者均检出ABCG5和（或）ABCG8基因突变，其中纯合突变4例（2例父母为近亲结婚），复合杂合突变4例（3例父母非近亲结婚）。ABCG5突变5例（R446X 4例、Q251X 4例）；ABCG8突变3例（Q298X、A519P、R263Q各1例），其中A519P尚未报道。所有检出的基因突变中，无义突变9例、错义突变2例、可变剪切位点突变1例（表2）。

**表2:** 8例植物固醇血症患者的遗传学特征和血小板形态学特点

例号	基因突变	遗传模式	父母近亲结婚	MPV（fl）	LPR（％）	MPD（µm）
1	ABCG5: exon10: c.C1336T: p. R446X；ABCG5: exon6: c.C751T: p. Q251X	复合杂合	否	18.3	79.5	3.5（1.1～11.2）
2	ABCG5: exon10: c.C1336T: p. R446X	纯合	是	16.8	72.2	2.9（1.0～10.1）
3	ABCG5: exon10: c.C1336T: p. R446X；ABCG5: exon6: c.C751T: p. Q251X	复合杂合	否	较多巨大血小板	/	4.1（2.0～8.7）
4	ABCG5: exon10: c.C1336T: p. R446X；ABCG5: exon6: c.C751T: p. Q251X	复合杂合	否	15.0	70.7	3.9（1.6～8.1）
5	ABCG8: exon6: c.C892T: p. Q298X	纯合	是	17.7	84.2	3.8（1.1～9.0）
6	ABCG8: exon11: c.G1555C: p. A519P	纯合	/	16.3	67.2	3.7（1.2～8.0）
7	ABCG5: exon7: c.904+1G>A；ABCG5: exon6: c.C751T: p. Q251X	复合杂合	/	较多巨大血小板	/	4.1（1.9～15.0）
8	ABCG8: exon6: c.G788A: p.R263Q	纯合	/	较多巨大血小板	/	5.2（1.6～21.0）

**注** MPV：平均血小板体积；LPR：大血小板比例；MPD：平均血小板直径；/：不详。生物参考值：MPV 8.0～12.0 fl，LPR 13.0％～43.0％

三、外周血涂片和骨髓象

8例患者外周血涂片中均可以看到红细胞形态异常，以口形红细胞和靶形红细胞为主，细胞大小不等。另外还可见到大血小板和巨大血小板（图1）。2例患者骨髓象可见有核细胞增生活跃，幼红细胞比值增高、晚幼红比值偏高，巨核细胞数量正常或增多，产血小板巨核细胞数量不少，易见大血小板。

**图1:**
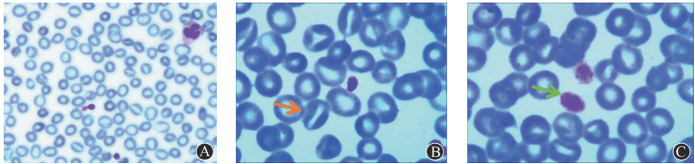
植物固醇血症患者（例1）外周血涂片（瑞士吉木萨染色） **A** 红细胞大小不等，可见较多口型红细胞（×200）；**B** 箭头所示为口型红细胞（×1000）；**C** 箭头所示为巨大血小板（×1000）

四、血小板体积

为了进一步研究植物固醇患者外周血血小板体积大小的特点，我们重点观察了8例患者血常规中平均血小板体积（MPV）和大血小板比例（LPR）两个参数，以及外周血涂片中血小板直径相关参数，并与健康对照和ITP患者相比较，见表2、表3。健康对照组平均血小板计数为（230.35±41.82）×10^9^/L、MPV（10.27±0.82）fl、LPR（27.86±5.64）％、MPD（2.6±0.4）µm。8例患者中有3例因血小板体积巨大、血细胞自动计数仪无法计算出MPV和LPR，镜下复检见到较多巨大血小板，其余5例MPV和LPR明显高于健康对照和ITP患者（*P*<0.05）；外周血涂片中，患者血小板直径差异较大（最大直径21 µm，最小直径1.0 µm），MPD为（3.9±0.6）µm，明显高于健康对照组和ITP组（*P*<0.05）；直径4～8 µm大血小板占比（45.00±13.15）％，明显高于健康对照组和ITP组（*P*<0.05）；7例患者可以看到直径大于8 µm的巨大血小板，而健康对照组和ITP患者无巨大血小板。

**表3:** 植物固醇血症患者、健康对照组与原发免疫性血小板减少症（ITP）患者的血小板参数比较

组别	例数	PLT（×10^9^/L）	MPV（fl）	LPR（％）	D<4 µm（％）	D 4～8 µm（％）	D>8 µm（％）	MPD（µm）
植物固醇血症	8	76.50±31.78^ab^	16.82±1.28^ab^	74.7±6.9^ab^	52.8±15.0^ab^	45.0±13.1^ab^	1.4±0.4^ab^	3.9±0.6^ab^
ITP	20	29.86±14.96^a^	12.23±2.05^a^	46.4±11.1^a^	91.5±3.1^a^	8.5±1.1^a^	0	3.1±1.1^a^
健康对照	30	225.35±36.83	10.32±0.75	25.4±4.5	95.4±3.1	4.6±0.4	0	2.6±0.4

**注** MPV：平均血小板体积，LPR：大血小板比例，D：血小板直径，MPD：平均血小板直径；生物参考值：PLT（125.00～350.00）×10^9^/L，MPV 8.00～12.00 fl，LPR 13.0％～43.0％；^a^与健康对照组比较，*P*<0.05，^b^与ITP组比较，*P*<0.05

五、依折麦布治疗前后的变化

5例患者接受低植物固醇饮食和依折麦布10 mg/d口服治疗。治疗3个月后，血中谷固醇和菜油固醇含量下降，HGB和血小板计数上升、外周血涂片中MPD下降。3例患者完成了6个月的治疗。

## 讨论

自1974年由Bhattacharyya等[6]首次报道，其后的30年间，植物固醇血症作为一种遗传代谢性疾病被人们逐渐认识，其主要表现为皮肤和肌腱黄色瘤、动脉粥样硬化、早发型冠心病、关节疼痛等。2005年Rees等[1]首次报道该疾病可伴有血液系统改变。随后1年，我国学者苏雁华等[2]首次报道了以血小板减少和溶血性贫血为首发临床表现的植物固醇血症家系，多伴有脾大；外周血涂片可见口形和靶形红细胞，巨大血小板，红细胞渗透脆性明显增加[2],[7]。过量的植物固醇在血浆和不同组织中积聚，可造成不同组织和器官损伤，其首发临床表现异质性极高，可能到不同专科就诊；加之该病发病率低，往往被误诊为不同专科的常见病（家族性高胆固醇血症、心血管疾病、类风湿关节炎、骨关节病、ITP、Evans综合征等）并接受不恰当的治疗。从症状出现到疾病被确诊，其平均延误时间可达28.8年[8]。本研究中8例患者全部以血小板减少为首发症状，不伴贫血或伴轻度贫血，大部分患者有脾大甚至经历脾切除治疗，部分患者胆固醇、甘油三酯、LDL-C升高，少部分患者有动脉粥样硬化、脂黄瘤和关节疼痛，没有冠状动脉疾病。本组病例临床特点与文献[8]–[9]报道相似。大部分患者都误诊为ITP，接受糖皮质激素、达那唑甚至脾切除等错误治疗，确诊时年龄最大为66岁，诊断延误时间长达50年。

巨大血小板是植物固醇血症的特征性血液学表现之一，其可能机制为植物固醇堆积导致巨核细胞分界膜系统分化障碍所致，并非由ABCG5/ABCG8基因突变直接引起[10]–[12]。在早期对遗传性血小板减少性疾病（IT）的研究中，科学家们发现描述血小板大小的相关参数是区分遗传性大血小板减少症和ITP这一类获得性血小板减少症的重要信号。早在2009年意大利一项单中心研究显示，当MPV大于12.4 fl或MPD大于3.3 µm时，鉴别遗传性大血小板减少症和ITP的敏感性和特异性都高于80％[13]；这一临界值在2013年的多中心大样本研究中得以进一步证实[14]。2014年《BLOOD》报道了一项全球多中心研究，纳入376例（19种）IT、87例ITP患者和55位健康志愿者，发现通过分析外周血涂片中MPD可以有效区分拥有巨大血小板的IT（MYH9-RD、BSS）和ITP以及其他类型IT；当MPD大于3.74 µm时，敏感性和特异性都高于85％；再结合外周血涂片中直径大于3.9 µm（约红细胞直径的一半）的血小板比例超过39.8％，鉴别的敏感性和特异性甚至高达98％[15]。为了解植物固醇血症患者血小板大小的具体特征，我们比较了该类患者、ITP患者和健康对照三组间血小板大小相关参数的差异。5例患者血常规中MPV和LPR明显大于健康对照和ITP患者，8例患者外周血涂片中MPD明显高于健康对照和ITP患者，并且直径大于4 µm的血小板比例超过40％，此特征与MYH-9突变相关疾病（MYH9-RD）和Bernard-Soulier综合征（BSS）的血小板形态特征相似。说明关注血常规中MPV、LPR这两个参数，加上血小板形态特征的观察，结合红细胞特征性改变（口形和靶形红细胞）有助于该病和ITP相鉴别。与此同时，该病与同样拥有巨大血小板的IT（如MYH9-RD、BSS等）的鉴别需要综合分析：MYH9-RD外周血除了出现巨大血小板减少之外，还可能出现中性粒细胞包涵体，红细胞形态一般正常，或者出现中心淡染区扩大的缺铁性贫血的表现；部分患者会伴有听力损伤或者肾炎表现[16]。BSS患者典型表现除了血小板巨大和血小板减少外，还伴随出血时间延长、瑞斯托霉素不能诱导血小板聚集和血小板膜表面GPIβ和GPIX表达下降，一般没有溶血性贫血和脾大的表现[17]。基因检测发现ABCG5/ABCG8致病突变以及血中植物固醇含量升高是确诊植物固醇血症的金标准。

ABCG5基因和ABCG8基因并排位于人类染色体2p21上，两者均含有13个外显子，分别编码固醇外排转运蛋白sterolin-1和sterolin-2。这两种蛋白形成二聚体定位于小肠上皮细胞和肝细胞，将细胞中固醇物质（胆固醇和植物固醇）排出到肠腔和胆汁中，从而减少健康人对固醇的吸收[3]。既往研究报道在东亚患者中ABCG5基因突变比例更高，其与ABCG8基因突变的发生比率约为3:1,而白种人患者多为ABCG8基因突变[18]。迄今为止，在植物固醇血症患者中已经报道了多达80种不同的变异，38个影响ABCG5，42个变异位于ABCG8基因[18]。无义变异和错义变异是最常见的变异类型，而剪接位点变异相对少见[18]。患者的突变分布与以上特点相符合。目前还没有明确的证据表明植物固醇血症的基因型与表型间的相关性,然而在已报道的引起植物固醇血症的变异中，有24个与大血小板减少相关[19]。本研究中，ABCG5的无义突变R446X和Q251X、ABCG8的错义突变R263Q均被报道与大血小板减少表型相关，其中ABCG5的R446X为该临床表型的热点突变位点。Xia等[18]对55例儿童患者的研究指出儿童早发性脂黄瘤患者双等位氨基酸改变往往位于固醇转运蛋白sterolin-1和sterolin-2的转运体结构域，该结构域缺陷可导致严重的高胆固醇血症，而跨膜结构域缺陷可能导致较轻的表型。本研究为成人患者，未观察到同样现象，3例有脂黄瘤的患者黄瘤出现时间较晚。因此，植物固醇血症患者的基因型-表型的关系需要更大样本、不同年龄阶段的数据来阐明。

控制饮食中植物固醇的摄入和药物降低血浆植物固醇水平是目前植物固醇血症的主要治疗手段。低植物固醇饮食可使血浆植物固醇水平降低约30％[20]。消胆胺是一种胆汁酸螯合剂，通过破坏胆汁酸的肠肝循环并抑制回肠中胆汁酸的重吸收，可以减少血浆中约45％的植物固醇，但由于其消化道耐受性差目前已较少应用[20]–[21]。固醇吸收抑制剂依折麦布可与固醇吸收转运蛋白尼曼-匹克C1样1（Niemann-Pick C1-like l, NPC1L1）结合，抑制植物固醇和游离胆固醇在肠上皮细胞的吸收。多项研究表明，依折麦布10 mg/d可显著降低血清植物固醇水平，改善动脉粥样硬化程度，缩小脂黄瘤体积，改善贫血和大血小板减少症[18],[22]–[25]。本研究中5例患者在低植物固醇饮食和依折麦布治疗后植物固醇浓度下降、血液学异常得到改善，血小板体积亦见缩小。

综上所述，植物固醇血症是一种罕见的遗传代谢性疾病，首发临床表现异质性高，极易误诊。以血小板减少为首发症状的患者，关注血小板大小相关参数，结合外周血巨大血小板和红细胞特征性改变（口形和靶形红细胞）有助于早期识别该病。虽然该病无法根治，但可通过控制饮食中植物固醇摄入和药物降低血浆植物固醇水平，达到有效延缓疾病发展、减少并发症的发生的目标。
